# Distribution of Microbes and Drug Susceptibility in Patients with Diabetic Foot Infections in Southwest China

**DOI:** 10.1155/2018/9817308

**Published:** 2018-08-05

**Authors:** Mingxia Wu, Hang Pan, Weiling Leng, Xiaotian Lei, Liu Chen, Ziwen Liang

**Affiliations:** Department of Endocrinology, Southwest Hospital of Third Military Medical University (Army Medical University), Chongqing 400038, China

## Abstract

**Objective:**

To investigate the microbial distribution and drug susceptibility among diabetic foot ulcers (DFUs) with different Wagner grades and between acute and chronic DFUs*. Methods*. We enrolled 428 DFU patients who were hospitalized and treated in the Southwest Hospital. We collected deep ulcer secretion for microbial culture and drug susceptibility tests and analyzed the results. We reexamined 67 patients with poor anti-infection efficacy and analyzed microbial species. *Results*: The 354 positive samples included 201 cases (56.8%) of single-pathogen infections and 153 cases (43.2%) of multiple-pathogen infections before antibiotic therapy. A total of 555 strains were cultivated, including 205 (36.9%) strains of gram-positive organisms (GPOs), 283 (51.0%) gram-negative bacilli (GNB), and 67 (12.1%) fungal strains. In terms of distribution, patients with different Wagner grades had different bacterial composition ratios (*P* < 0.01). Patients with Wagner grades 3–5 mainly had GNB. The specimens from chronic ulcer wounds were primarily GNB (54.2%), whereas fungi accounted for 14.4% of the infections; the distribution was significantly different from that of acute ulcers (*P* < 0.01). The susceptibility tests showed that the *Staphylococcus* genus was more susceptible to vancomycin, linezolid, and tigecycline. Tobramycin was the most effective drug (97%) for the treatment of *Escherichia coli*, followed by ertapenem (96.4%), imipenem (93.5%), and cefotetan (90%). Most of the remaining GNB were susceptible to antibiotics such as carbapenems, aminoglycosides, fluoroquinolones, ceftazidime, cefepime, and piperacillin-tazobactam (>63.2%). After antibiotic therapy, the positive rate of microbial culture was 52.2%, and the proportion of GNB and fungi increased to 68.9% and 20%.

**Conclusion:**

The distribution and types of bacteria in diabetic foot infection (DFI) patients varied with the different Wagner classification grades, courses of the ulcers, and antibiotic therapy. Multidrug resistance were increased, and the clinical treatment of DFIs should select the most suitable antibiotics based on the pathogen culture and drug susceptibility test results.

## 1. Introduction

Diabetes is prevalent worldwide. Diabetic foot disease is one of the most difficult to treat complications of diabetes and has become an important cause of nontraumatic amputation. The probability of diabetic patients suffering from diabetic foot ulcers (DFUs) during their lifetime can reach 25% [1], and the amputation rate for China's DFU patients is up to 21.5% [2]. The risk of a DFU complicated by a diabetic foot infection (DFI) is high [3]. DFIs not only extend the average length of the hospital stay, resulting in a huge economic burden [4] but also increase the risk of amputation [5], which seriously affects the quality of life and life expectancy of patients with diabetes. Control of DFIs in a timely and effectively manner has become an urgent problem for clinicians. Studies from different countries have revealed different DFI-related microbial compositions and drug susceptibilities [6–8], and the ratios of patients associated with multidrug resistance (MDR), methicillin-resistant *Staphylococcus* (MRS), and extended-spectrum *β*-lactamase (ESBL) bacterial infections have increased every year, suggesting that administration of empirical anti-infective regimens will increase the chances of treatment failure. China has a large population of DFI patients with a vast geographical distribution and significant variations in the types of bacterial infections found in DFI wounds from different regions. However, studies on this aspect are rare. In this study, we aimed to retrospectively analyze the pathogen culture and drug susceptibility test results for DFI patients in southwest China to help clinicians choose a more appropriate standard antibiotic treatment for DFIs.

## 2. Patients and Methods

A total of 428 DFI patients who were hospitalized from January 2014 to June 2017 in the Diabetic Foot Center at the Southwest Hospital of the Third Military Medical University, which is a large tertiary grade A hospital in southwest China, were enrolled in this study. Diabetic foot secretion samples were collected for the microbial culture and drug susceptibility tests. Before DFI patients were treated with antibiotics, they should undergo debridement with normal saline. After removing surface carrion and exudate, deep ulcer secretion should be taken with sterile cotton swab, kept by sterile tube, and sent to microbiology lab of laboratory medicine quickly for anaerobic bacteria, aerobic bacteria, fungal culture, and drug susceptibility test. Bacterial drug resistance was determined based on the antimicrobial susceptibility test guidelines published by the Clinical and Laboratory Standards Institute (CLSI). MDR strains were determined according to the interim standard definition of acquired resistance published by Magiorakos et al. [9]. We also collected basic information, diabetes-related complications, and other information from the DFI patients. In some patients, there was no significant improvement or continuous aggravation in the wound after >7days of antibiotic therapy. The above method was used again to examine the deep ulcer secretion and to count the species of bacteria. The DFUs were graded using the Wagner classification system. The ulcer disease course was staged based on acute and chronic wound staging; chronic ulcers referred to those with no improvement after 4 weeks of treatment or those not cured within 8 weeks [10].

All data were analyzed using the SPSS 19.0 software. The measurement data were expressed as the mean ± standard deviation ($\bar{x}\pm s$) and analyzed using an independent *t*-test. The counting data were analyzed with the chi-square test with a significance level of *α* = 0.05.

## 3. Results

### 3.1. Patient Information

In this study, we included the tissue culture results from a total of 428 DFI cases, including 273 (63.8%) male patients and 155 (36.2%) female patients. The patients were aged between 25 and 94 years, with an average age of 65.1 ± 11.9 years, and 292 (68.3%) patients were aged 60 years and older. The average length of hospital stay varied with the different Wagner grades and wound stages. The duration of hospitalization was significantly longer for patients with chronic ulcer wounds than for patients with acute ulcer wounds (*t* = −2.917, *P* = 0.004). There were 354 (82.7%) DFI patients with vascular disease, 346 (80.8%) with peripheral neuropathy, 180 (42.1%) with renal lesions, and 200 (46.7%) with retinopathy. Of the 388 patients, 90 (23.2%) had good glycemic control (HbA1c ≤ 7%), 59 (15.2%) had fair glycemic control, and 239 (62.6%) had poor glycemic control (Table 1).

### 3.2. Microbial Culture

Before antibiotic therapy, a total of 354 of the 428 samples applied for testing were positive cases, for a positive rate of 82.7%. The positive rate of Wagner grades 2–5 was significantly higher than that of Wagner grade 1 (*X*^2^ = 33.911, *P* ≤ 0.001). There were 201 cases (56.8%) with single-pathogen infections and 153 cases (43.2%) with multiple-pathogen infections (microbial strain numbers ≥ 2). Samples from Wagner grade 2-3 ulcers mainly had single-pathogen infections, whereas those from Wagner grade 4-5 ulcers mainly had multiple-pathogen infections; additionally, differences in the microbial distribution were observed between the different Wagner grades (*X*^2^ = 11.101, *P* = 0.025). A total of 555 strains were cultivated, including 205 (36.9%) gram-positive organisms (GPOs), 283 (51.0%) gram-negative bacilli (GNB), and 67 (12.1%) fungal strains. Samples from Wagner grade 3–5 ulcers mainly had GNB, with differences in the microbial distribution between different Wagner grades (*X*^2^ = 25.278, *P* = 0.001). *Staphylococcus aureus* was the most common GPO in the ulcers with different Wagner grades. Ulcers with Wagner grade 3 had the highest incidence rates of MDR, MRS, and ESBL at 32.4%, 47.1%, and 40%, respectively. The proportion of *Enterococcus* increased gradually with the higher Wagner grades. The most common gram-negative bacteria in Wagner grade 2–5 ulcers were *Klebsiella* (14.7%), *Escherichia coli* (18.7%), *Pseudomonas aeruginosa* (17.7%), and *Proteus* (31.6%) (Table 2).

**Figure 1:**
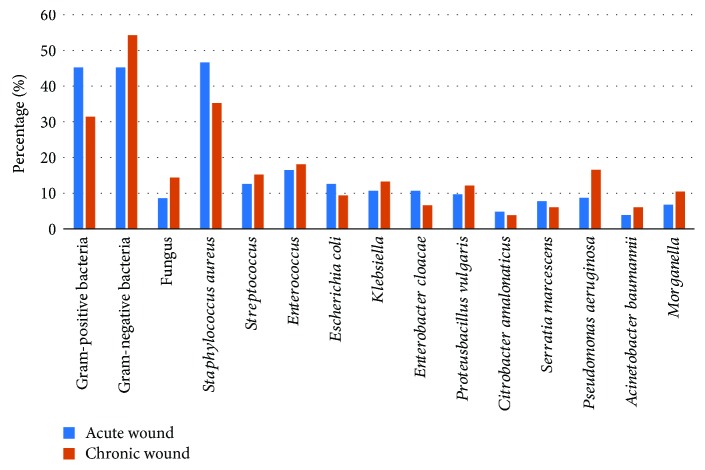
The distribution of pathogenic bacteria was detected in DFI with different duration of ulcer.

The positive rates for the GPO or GNB culture results in the acute ulcer wounds were both 45.2%. *Staphylococcus aureus* was still the most common GPO (46.6%), and the GNB mainly consisted of *Escherichia coli* (12.6%). The specimens from chronic ulcer wounds mainly had GNB (54.2%), and fungi accounted for 14.4% of the infections. Comparing the acute ulcer wounds with the chronic ulcer wounds showed significant differences in the microbial composition (*X*^2^ = 184.449, *P* ≤ 0.001), with *Pseudomonas aeruginosa* (16.6%) the most common GNB (Figure 1).

We reexamined the deep ulcer secretions of 67 patients with poor anti-infection efficacy (>7 days). 35 specimens training result was positive, positive rate of 52.2%. A total of 45 strains were cultured, mainly with monomicrobial infection (68.4%), among which 11.1%, 68.9%, and 20% were gram-positive coccus, gram-negative bacilli and fungus, respectively. *Escherichia coli* (19.4%), *Pseudomonas aeruginosa* (19.4%), and *Proteusbacillus vulgaris* (16.1%) were the main gram-negative bacilli (Table 2).

### 3.3. Drug Susceptibility Test

**Figure 2:**
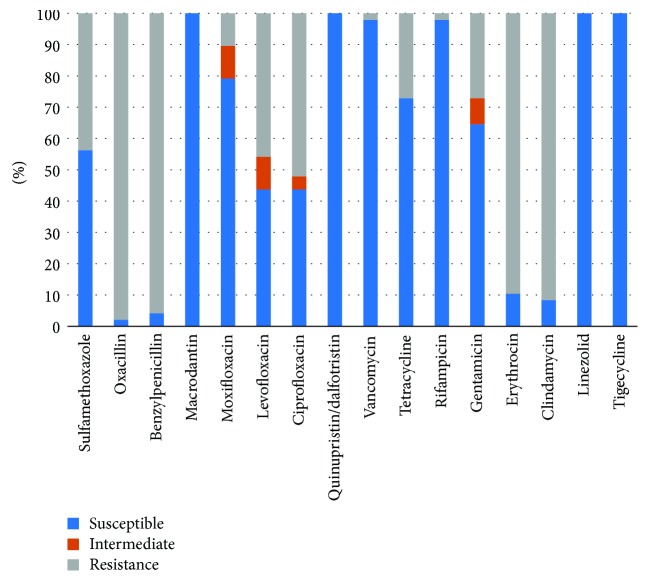
The susceptible pattern of MRS from diabetic foot patients.

The *Staphylococcus* genus was more susceptible to vancomycin, linezolid, and tigecycline, with only 1 case of vancomycin-resistant *Staphylococcus epidermidis*. A total of 48 MRS strains of the *Staphylococcus* genus were identified from the culture and drug susceptibility tests. The MRS strains were more susceptible to linezolid (100%), tigecycline (100%), and vancomycin (97.9%), followed by moxifloxacin (79.2%), and showed poor susceptibility to clindamycin (8.3%) and erythromycin (10.4%) (Figure 2). There was 1 case of vancomycin-resistant *Enterococcus faecalis* among the *Enterococcus* strains, and *Enterococcus faecalis* was most susceptible to tigecycline (100%) and ampicillin (100%), followed by vancomycin (96.6%), penicillin G (96.6%), and linezolid (86.2%). The susceptibilities of *Enterococcus faecium* to vancomycin, linezolid, and tigecycline were all 100% (Table 3).

**Figure 3:**
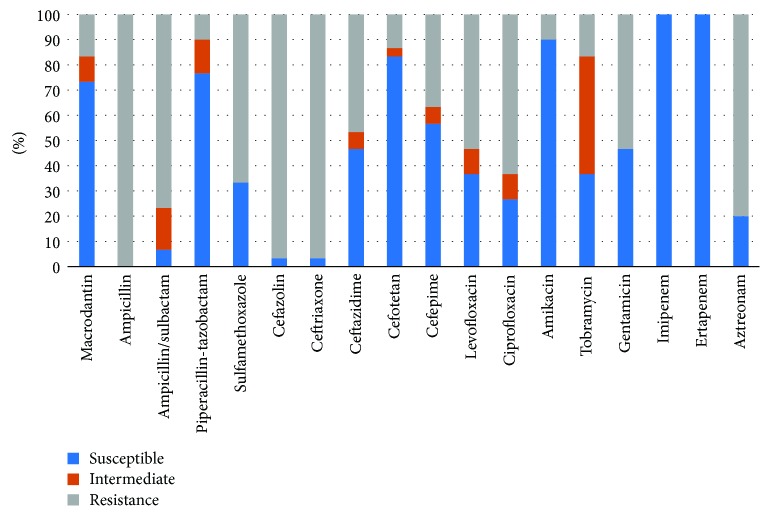
The susceptible pattern of product ESBL bacteria from diabetic foot patients.

Tobramycin was the most effective drug (97%) for the treatment of *Escherichia coli*, followed by ertapenem (96.4%), imipenem (93.5%), and cefotetan (90%), but *Escherichia coli* had poor susceptibility to amikacin, gentamicin, and levofloxacin (all <50%). Most of the remaining GNB were susceptible to antibiotics such as carbapenems, aminoglycosides, fluoroquinolones, ceftazidime, cefepime, and piperacillin-tazobactam (>63.2%) (Table 4). In addition, this study cultured 30 ESBL strains, which showed high susceptibilities (100%) to imipenem and ertapenem, followed by amikacin (90%), cefotetan (83.3%), and piperacillin-tazobactam (76.7%), and were less susceptible to levofloxacin (36.7%) and ciprofloxacin (26.7%) (Figure 3).

**Figure 4:**
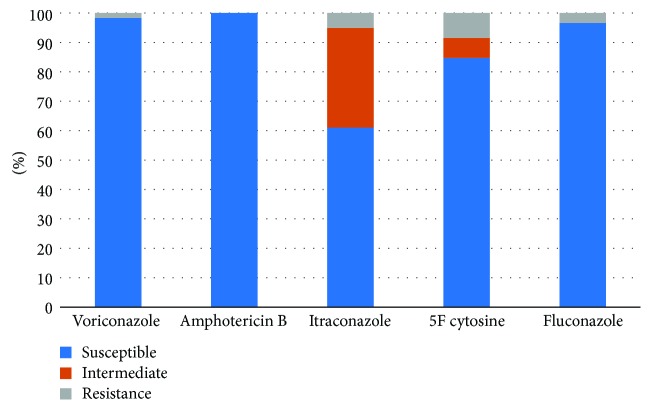
The susceptible pattern of Fungus from diabetic foot patients.

The proportion of fungi was only 12.1%. The proportion of fungi was increased in Wagner grade 3–5 ulcers, but the difference was not significant (*X*^2^ = 3.954, *P* = 0.412). The susceptibilities of the fungi to voriconazole, amphotericin B, itraconazole, 5-fluorocytidine, and fluconazole were 98.3%, 100%, 62.1%, 84.7%, and 96.8%, respectively (Figure 4).

## 4. Discussion

Consistent with most studies [11, 12], the DFI patients were often elderly males with multiple complications, possibly due to the burdens of life and exercise habits. Most of the patients had poor glycemic control. The patients with Wagner grades 2–5 had significantly longer hospital stays than did the grade 1 patients, and patients with chronic DFUs had longer hospital stays than those with acute DFUs (*t* = −2.704, *P* < 0.05).

Our results indicated that the DFI-causing bacteria were dominated by GNB (51%), which differed from the results of the survey performed in southern China from 2009 to 2014 [13] in which GPOs accounted for 54% of the infections. This finding suggests that different regions may have different dominant DFI pathogens or that GNB may have replaced GPOs as the main pathogens in Chinese DFI patients. We also noted that different Wagner grades and changes in the ulcer course led to different bacterial distributions and types. Patients with Wagner grades 1-2 mostly had GPOs, whereas those with grades 3–5 mostly had GNB; additionally, acute wounds had similar ratios of GPOs and GNB, but chronic infections mostly had GNB. After antibiotic therapy, the positive rate of bacterial culture decreased significantly, and the proportion of GNB and fungi increased significantly. Studies have shown that GNB infections are positively correlated with amputation and negatively correlated with DFU healing [14], suggesting that GNB infections are a serious DFI warning. A study from Pakistan from 2013-2014 [15]showed that mixed infections accounted for the majority (56.9%), whereas the results of our study showed predominantly single-pathogen infections (56.8%). Further analysis showed that patients with Wagner grades 1–3 mainly had single-pathogen infections, whereas those with Wagner grades 4-5 mainly had multiple-pathogen infections, indicating that multiple-pathogen infections were also a sign of severe DFIs.

*Staphylococcus aureus* is the most common GPO. However, the percentage of *Staphylococcus aureus* decreased with the increased Wagner level and prolonged duration of the ulcer, whereas the proportion of *Enterococcus* gradually increased. Agudelo Higuita and Huycke indicated that enterococci often appeared in patients with low immunity [16] and could participate in the formation of biofilms [17]. Biofilms can act as a virulence factor to cause treatment failure, suggesting that *Enterococcus* infection should be taken seriously. Studies from Mexico [18] showed that the resistance rate of *Staphylococcus aureus* to vancomycin was as high as 49%. Our results suggested that most GPOs, including *Staphylococcus aureus*, were susceptible to vancomycin, linezolid, and tigecycline and were resistant to penicillin G, erythromycin, and clindamycin. These findings were different from the observations from Bravo-Molina et al. [19], which showed that fluoroquinolone antibiotics were the most susceptible antibiotics for GPOs. In this study, only *Staphylococcus aureus* showed good susceptibility to fluoroquinolone antibiotics (>80%), whereas the other GPOs showed low susceptibility.

In 1961, the first methicillin-resistant *Staphylococcus aureus* (MRSA) was found in the UK [20]. Today, various MDR strains have become epidemic strains worldwide. We identified 182 (32.8%) MDR strains from the 558 strains, including 51.1% of the GNB, which slightly differed from the results of a study from Tianjin, China [21]. We cultured 31 MRS strains, of which 17 were MRSA strains, accounting for 20% of the *Staphylococcus aureus* strains. Our result is consistent with the results from studies in Pakistan [15] and differs from the 78% of strains found by a study in Iran [22]. The MRS susceptibility test showed that the MRS strains were still highly susceptible to vancomycin, linezolid, and tigecycline but showed significantly reduced susceptibility to levofloxacin and ciprofloxacin, which are frequently used in clinical practices. This finding suggests that the occurrence of MRS will increase the risk of anti-infective treatment failure. Long-term (over 6 months) use of antibiotics, a long ulcer course, high blood pressure, anemia, and chronic osteomyelitis are all MRSA risk factors [23]. Clinicians should be alerted to the possibility of associated MRSA infections in patients with the above conditions and can select antibiotics capable of treating this type of pathogen. In addition, studies have shown that *Staphylococcus aureus* infection is more likely to cause the formation of bacterial biofilms in diabetic foot wounds [24], which reduces the susceptibility of bacteria to antibiotics. Genetic testing can determine whether *Staphylococcus aureus* is invasive [25]. Therefore, methods such as biofilm detection and genetic testing may be used as a new means of detection in the future to improve DFI assessment in clinical practice.

In contrast to the results of Gadepalli et al. [11], *Pseudomonas aeruginosa* accounted for the highest percentage of the GNB, followed by *Klebsiella* and *Escherichia coli*. Further analysis showed that the most common GNB types among patients with chronic wounds were *Pseudomonas aeruginosa*, *Klebsiella*, and *Proteus*, which accounted for 16.6%, 13.3%, and 12.2%, respectively; these results were similar to the findings of de Vries et al. [14]. The Enterobacteriaceae family showed the highest susceptibility to ertapenem and imipenem. Sugandhi and Prasanth [26] suggested that amikacin was the most susceptible antibiotic for the treatment of GNB. However, we found that the susceptibility of *Escherichia coli* to amikacin was only 46.9%. The susceptibilities of *Escherichia coli* to the commonly used levofloxacin and ciprofloxacin were also low (<40%), whereas the susceptibility of this bacterium to piperacillin-tazobactam, cefotetine, and tobramycin was higher (>87.9%). In contrast, *Klebsiella* was more susceptible to fluoroquinolones (>81.1%). Partially consistent with the results of studies on *Pseudomonas aeruginosa* from Pakistan [27], we found that this bacterium was less susceptible to ampicillin and was more susceptible to quinolone antibiotics. In China, *Pseudomonas aeruginosa* is fairly susceptible to aminoglycoside antibiotics (>84.6%), possibly because these antibiotics are less frequently used in China at present.

In this study, we found 30 strains of ESBL-producing *Enterobacter*, which accounted for 10.6% of the GNB. Most of these strains were derived from *Escherichia coli* and were resistant to most antibiotics; these strains showed the highest susceptibility to carbapenems (100%), followed by amikacin (90%), cefotetan (83.3%), and piperacillin-tazobactam (76.7%), and lower susceptibility to fluoroquinolones (<36.7%), which was consistent with the results from Bangladesh and Nepal [28, 29]. This study did not find carbapenem-resistant ESBL-producing bacteria. ESBL-producing bacterial infections increase the hospitalization rate of DFI patients and further reduce the choice of antibiotics [30]. For example, fluoroquinolone antibiotics, which are frequently used in our hospital, show a significantly reduced susceptibility to ESBL-producing bacteria, suggesting that we should perform drug susceptibility testing to select susceptible antibiotics for treatment.

In summary, different Wagner grades and changes in the course of ulcers led to different distributions of bacteria and different bacterial species. Wagner grades 4-5 and chronic ulcer wounds had high ratios of GNB infections and mixed infections. For patients at risk of infections with MDR, MRS, and ESBL-producing bacteria, clinicians should focus on the use of antibiotics for the treatment of these types of bacteria when conducting empiric therapy and should adjust the drugs according to the results of the drug susceptibility test and clinical treatment efficacy. In addition to actively applying appropriate antibiotic treatment, multidisciplinary management combined with foot pressure reduction, timely debridement, and lower extremity vascular intervention should be applied to increase the success rate of anti-infection treatment and to reduce the amputation rate.

## Figures and Tables

**Table 1:** Clinical and demographical variables.

Parameter	Values	Values (range or *n* (%))
Gender	Male	273 (63.8)
	Female	155 (36.2)
Age	<40 years	4 (0.9)
	40–50 years	39 (9.1)
	50–60 years	93 (21.7)
	60–70 years	122 (28.5)
	>70 years	170 (39.8)

Hospital stays (days)	Wagner grade 1	11.5 ± 6.1
	Wagner grade 2	19.3 ± 14.9
	Wagner grade 3	22.1 ± 17.4
	Wagner grade4	21.0 ± 17.3
	Wagner grade 5	22.3 ± 13.4
	Duration of ulcer ≤ 4 weeks	17.6 ± 12.6
	Duration of ulcer > 4 weeks	21.8 ± 17.8

Complication	Vascular diseases	354 (82.7)
	Neuropathy	346 (80.8)
	Nephropathy	180 (42.1)
	Retinopathy	200 (46.7)

HbA1c (%)	≤7% (good control)	90 (23.2)
	7.1–8% (fair control)	59 (15.2)
	8.1–10% (poor control)	109 (28.1)
	>10% (very poor control)	130 (33.5)

**Table 2:** The distribution of pathogenic bacteria was detected in DFI with different wagner grades.

Before antibiotic therapy, *n* (%)							After antibiotic therapy, *n* (%)
	Wagner grading						
	1	2	3	4	5	Total	
Total samples	36 (8.4)	114 (26.6)	155 (36.2)	105 (24.5)	18 (4.3)	428	67
Positive samples	20 (55.6)	101 (88.6)	119 (76.8)	98 (93.3)	16 (88.9)	354 (82.7)	35 (52.2)
Total strains	28	156	176	165	30	555	45
Monomicrobial infection	17 (47.2)	59 (58.4)	73 (61.3)	46 (46.9)	7 (38.9)	201 (56.8)	24 (68.6)
Polymicrobial infection	4 (11.1)	42 (41.6)	46 (38.7)	52 (53.1)	9 (61.1)	153 (43.2)	11 (31.4)
MDR	7 (3.8)	50 (27.5)	59 (32.4)	58 (31.9)	8 (4.4)	182 (32.8)	20 (57.4)
Gram-positive bacteria	18 (64.3)	73 (46.8)	62 (35.2)	45 (27.3)	7 (23.3)	205 (36.9)	5 (11.1)
*Staphylococcus aureus*	11 (61.1)	29 (39.7)	29 (46.8)	15 (33.3)	1 (14.3)	85 (41.5)	4 (80.0)
Other *Staphylococcus*	6 (33.3)	26 (35.6)	7 (11.3)	9 (20.0)	1 (14.3)	49 (23.9)	1 (20.0)
MRSA	1 (5.9)	5 (29.4)	8 (47.1)	3 (17.6)	0 (0)	17	4
MRS	2 (6.5)	17 (54.8)	2 (6.5)	9 (29.0)	1 (3.2)	31	0
*Streptococcus*	1 (5.6)	6 (3.8)	13 (21.0)	6 (13.3)	3 (42.9)	29 (14.1)	0
*Enterococcus*	0 (0)	10 (8.2)	11 (17.7)	13 (28.9)	2 (28.6)	36 (17.6)	0
Gram-negative bacteria	9 (32.1)	68 (43.6)	91 (51.7)	96 (58.2)	19 (63.3)	283 (51.0)	31 (68.9)
*Escherichia coli*	1 (11.1)	4 (5.9)	17 (18.7)	10 (10.4)	1 (5.3)	33 (11.7)	6 (19.4)
*Klebsiella*	1 (11.1)	10 (14.7)	12 (13.2)	9 (9.4)	3 (15.8)	35 (12.4)	4 (12.9)
Product ESBL	2 (6.7)	3 (10.0)	12 (40.0)	11 (36.6)	2 (6.7)	30	5
*Enterobacter cloacae*	1 (11.1)	5 (7.4)	5 (5.5)	11 (11.5)	1 (5.3)	23 (8.1)	1 (3.2)
*Proteusbacillus vulgaris*	0 (0)	2 (2.9)	11 (12.1)	13 (13.5)	6 (31.6)	32 (11.3)	5 (16.1)
*Citrobacter amalonaticus*	0 (0)	2 (2.9)	6 (6.6)	3 (3.1)	1 (5.3)	12 (4.2)	0
*Serratia marcescens*	1 (22.2)	2 (2.9)	5 (5.5)	10 (10.4)	1 (5.3)	19 (6.7)	1 (3.2)
*Pseudomonas aeruginosa*	2 (22.2)	8 (11.8)	9 (9.9)	17 (17.7)	3 (15.8)	39 (13.8)	6 (19.4)
*Acinetobacter baumannii*	1 (11.1)	5 (7.4)	3 (3.3)	6 (6.3)	0 (0)	15 (5.3)	1 (3.2)
*Morganella*	1 (11.1)	6 (8.8)	12 (13.2)	7 (7.3)	0 (0)	26 (9.2)	2 (6.4)
Fungus	1 (3.6)	15 (9.6)	23 (13.1)	24 (14.5)	4 (13.3)	67 (12.1)	9 (20.0)

**Table 3:** The susceptible pattern of gram-positive bacteria from diabetic foot patients.

	*S. aureus*	*S. haemolyticus*	*S. epidermidis*	*Enterococcus faecalis*	*Enterococcs faecium*	*Streptococcus*
Total strains	85	14	13	29	5	31
Sulfamethoxazole (%)	81.2	21.4	38.5	—	—	—
Oxacillin (%)	78.9	0	23.1	—	—	—
Ampicillin (%)	—	—	—	100	20	—
Benzylpenicillin (%)	5.9	0	0	96.2	—	—
Macrodantin (%)	98.8	100	100	88.9	0	—
Moxifloxacin (%)	95.3	57.1	84.6	69.6	0	—
Levofloxacin (%)	82.4	0	38.5	69.2	20	80.6
Ciprofloxacin (%)	81.2	0	23.1	57.1	0	—
Quinupristin/dalfotristin (%)	100	100	100	5	100	—
Vancomycin (%)	100	100	92.3	96.6	100	100
Tetracycline (%)	75.3	78.6	46.2	10.7	40	—
Rifampicin (%)	98.8	100	100	—	—	—
Gentamicin (%)	82.4	28.6	61.5	—	—	—
Erythrocin (%)	48.2	7.1	23.1	—	20	21
Clindamycin (%)	60	20	15.4	5.6	0	25.8
Linezolid (%)	100	100	100	86.2	100	100
Tigecycline (%)	100	100	100	100	100	—

**Table 4:** The susceptible pattern of gram-negative bacteria from diabetic foot patients.

	*E. coli*	*Serratia*	*Klebsiella*	*Enterobacter*	Proteusbacillus	*Pseudomonas*	*baumannii*	*Morganella*
Macrodantin (%)	79.3	10	35.1	55.6	—	2.6	14.3	0
Ampicillin (%)	21.9	—	5.4	0	55.6	0	0	0
Ampicillin/sulbactam (%)	25.8	—	48.6	0	73.7	5.9	50	0
Piperacillin-tazobactam (%)	87.9	100	94.6	84.2	100	74.4	80	96
Sulfamethoxazole (%)	31.3	94.7	67.6	61.9	52.6	5.3	64.3	40
Cefazolin (%)	22.2	5.3	27	18.8	—	0	0	3.8
Cefoxitin (%)	—	—	—	—	100	—	—	—
Ceftriaxone (%)	32.3	100	70.3	71.4	—	0	0	76.9
Ceftazidime (%)	62.5	100	89.2	81	100	87.2	66.7	88.5
Cefotetan (%)	90	—	94.6	9.1	—	0	0	100
Cefepime (%)	66.7	94.7	94.6	90.5	73.7	89.7	71.4	90.5
Levofloxacin (%)	39.4	94.7	83.8	75	73.7	89.5	73.3	92.3
Ciprofloxacin (%)	30.3	94.7	81.1	70	57.9	87.2	73.3	65.4
Amikacin (%)	46.9	78.9	75.7	76.2	63.2	87.2	93.3	53.8
Tobramycin (%)	97	100	94.6	93.3	88.9	89.5	—	88.5
Gentamicin (%)	46.9	94.7	78.4	95.2	68.4	84.6	93.3	53.8
Meropenem (%)	—	—	—	—	100	94.3	—	100
Imipenem (%)	93.5	89.5	100	100	68.4	82.1	73.3	—
Ertapenem (%)	96.4	100	100	94.7	100	—	—	100
Aztreonam (%)	48.5	84.2	83.8	76.2	84.2	—	22.2	100

## Data Availability

The data used to support the findings of this study are available from the corresponding author upon request.
